# Involvement of Hemopexin in the Pathogenesis of Proteinuria in Children with Idiopathic Nephrotic Syndrome

**DOI:** 10.3390/jcm10143160

**Published:** 2021-07-17

**Authors:** Agnieszka Pukajło-Marczyk, Danuta Zwolińska

**Affiliations:** Department of Pediatric Nephrology, Wroclaw Medical University, Borowska 213, 50-556 Wroclaw, Poland; danuta.zwolinska@umed.wroc.pl

**Keywords:** hemopexin, nephrotic syndrome, children

## Abstract

Hemopexin (Hpx) is considered a factor in the pathogenesis of idiopathic nephrotic syndrome (INS). The aim of the study was to evaluate the serum and urine values of Hpx (sHpx and uHpx) in children with INS, analyze the role of Hpx, and assess its usefulness as a marker of the disease course. 51 children with INS and 18 age-matched controls were examined. Patients were divided into subgroups depending on the number of relapses (group IA—the first episode of INS, group IB—with relapses) and according to method of treatment (group IIA treated with gluco-corticosteroids (GCS), group IIB treated with GCS and other immunosuppressants). Hpx concentrations were determined by enzyme-linked immunosorbent assay (ELISA). sHpx and uHpx values in relapse were elevated in the whole INS group versus controls (*p* < 0.000). In remission their levels decreased, but still remained higher than in the control group (*p* < 0.000). In group IB uHpx levels were increased during remission as compared to group IA (*p* < 0.006). No significant impact of immuno-suppressants on sHpx was observed, but uHpx excretion in group IIA was higher in relapse (*p* < 0.026) and lower in remission (*p* < 0.0017) as compared to group IIB. The results suggest the role of Hpx in the pathogenesis of INS. Hpx may be a useful indicator for continuation of treatment, but it requires confirmation by further controlled studies.

## 1. Introduction

Various factors can damage the glomerular filtration barrier and trigger nephrotic proteinuria. The child’s age at the time of the manifestation of symptoms allows the diagnostic process to be directed in search of the disease etiology. Up to 1 year of age, nephrotic syndrome is the result of mutations in genes encoding podocyte proteins or may be associated with congenital infections such as syphilis or toxoplasmosis [1]. Podocyte function may also be impaired in the course of lysosomal diseases, which should be considered in the diagnostic procedure, especially in the case of the development of nephropathy in young children [2]. In older children, nephrotic syndrome may develop in the course of autoimmune inflammatory diseases, such as lupus erythematosus, acute poststreptococcal glomerulonephritis, IgAN (IgA Nephropathy) or IgAVN (Immunoglobulin (Ig)A vasculitis nephritis) [3,4,5].

Idiopathic nephrotic syndrome (INS) is the most common form of podocytopathy in children. It accounts for over 90% of cases in the group between 1 and 10 years of age and about 50% in the group of children over 10 years of age. The incidence is estimated at about 16 cases per 100,000 of the pediatric population and at two–seven new cases per 100,000 children under 15 years of age. The diagnostic criteria are massive proteinuria above 50 mg/kg/day, hypoalbuminemia (<2.5 g/L) and edema [6,7]. These symptoms are accompanied by hyperlipidemia. Minimal change disease (MCD) is the most common morphologic feature of this syndrome (approximately 85% of cases), followed by focal segmental glomerulosclerosis (FSGS) and mesangial proliferative glomerulonephritis (MPGN). In each of these forms, there is the effacement of podocyte foot processes and structural disorganization of the glomerular filtration barrier (GFB). While the vast majority of patients (80–90%) respond well to gluco-corticosteroids (GCS), primary steroid resistance is observed in approximately 10%, mainly in FSGS patients, with a poorer prognosis for renal survival [8,9]. The course of INS is characterized by periods of remissions and relapses, usually induced by upper respiratory tract infections.

The pathogenesis of nephrotic proteinuria in MCD is complex and still not fully elucidated, as evidenced by the emergence of new hypotheses [10]. The earliest, from the 1970s, suggested that circulating protein permeability factors released by dysfunctional T lymphocytes were responsible for the development of proteinuria in MCD [11]. Various candidate cytokines, whose elevated levels were observed in the serum and urine of children with recurrent MCD, were considered. Out of these, IL-8 and IL-13 were found to be the most likely pathogenic factors. However, it should be emphasized that the reported results of many studies were inconclusive [12,13,14].

A more recent theory, the “two-hit” theory, posits that the process of podocyte damage is more complex and partially combines the earlier hypotheses [15,16]. The first hit is the stimulation of podocytes by T-linked cytokines, bacterial or viral fragments, allergens or other factors, resulting in increased expression of CD80 (B7-1). Induction of CD80 leads to podocyte damage and increased permeability to proteins. If normal podocyte autoregulation is maintained, T-regulatory cells (T-reg) prevent this phenomenon, with the involvement of the CTLA-4 molecule, IL-10 and TGF-beta [17]. If these mechanisms fail, permanent overexpression of CD80 occurs, resulting in the full-blown MCD. In contrast, achieving remission after rituximab, a monoclonal antibody directed against CD-20, points to the involvement of B lymphocytes in INS development [18]. The role of circulating factors in the pathogenesis of proteinuria in INS remains of interest [19,20,21,22,23].

Hemopexin (Hpx) is a circulating plasma β-1 glycoprotein with a molecular weight of 60 kDa, encoded by a gene located on chromosome 11 (pp. 15.4–15.5) [24,25,26]. It is synthesized mainly in hepatocytes as a single polypeptide chain. Its spatial structure is determined by two disulfide bonds connecting structurally related domains (C- and N-terminal) and binding the heme moiety [27]. Hpx plays a role in iron homeostasis by binding heme released into serum and then transporting it to the liver where it is disintegrated [28,29]. The half-life of Hpx in serum is 7 days. Low concentrations may be a marker of hemolysis severity, whereas its absence may indicate either insufficient synthesis in the course of chronic liver diseases or severe malnutrition. Under physiological conditions, the mean urinary Hpx concentration is 2 mg/L, and its increase has been observed in diabetes, when glomerular proteinuria develops [30,31]. Increased levels of Hpx have also been shown in inflammatory psychiatric disorders, cancer, and neuromuscular diseases [32,33,34].

Hpx has serine protease activity. It exhibits anti- and pro-inflammatory effects and inhibits multinuclear granulocyte necrosis and cell adhesion [23]. In recent years, the effect of Hpx as a circulating factor on GFB permeability and the development of proteinuria has been postulated.

## 2. Aim of the Study

The aim of the study was to evaluate the serum and urine Hpx concentration in children with INS as a pathogenic factor and to determine its usefulness as a predictor of disease severity.

## 3. Material and Methods

The study group consisted of 69 children, including 51 children with INS (INS group), 19 girls and 32 boys, ranging in age from 1.25–18 years (mean age 8.86 ± 5.2 years). The diagnosis of INS was established according to ISKDC criteria [35]. Steroid sensitivity was defined as the achievement of remission during the first 4 weeks of GCS treatment, and steroid dependence as the occurrence of at least two relapses during the period of steroid dose reduction or within 2 weeks after GCS cessation. Remission was defined as the absence of protein in urine for at least 3 consecutive days.

The group of patients with INS was further divided into subgroups: group IA consisted of 20 children (5 girls, 15 boys; mean age 5.90 ± 4.91 years), with the first occurrence of INS, and group IB consisted of 31 children (14 girls, 17 boys; mean age 10.31 ± 4.81 years), with relapses (from 2–16 relapses, mean 11.8).

Additionally, in order to assess the severity of the course of INS, a second division was made according to the applied treatment. Group IIA included 26 children (7 girls, 19 boys; mean age 5.59 ± 4.04 years), treated only with GCS. In relapse standard doses of prednisone (2 mg/kg/day) were used, and in individual cases pulses of methylprednisolone 0.5 g/dose were also required. Group II B included 22 children (9 girls, 13 boys; mean age 12.31 ± 4.06 years) treated with GCS and corticosteroid-sparing agents: cyclosporin (CsA), mycophenolate mofetil (MMF), and azathioprine (AZA). The distribution of administered immunosuppressive drugs in this subgroup was as follows: 17 children—CsA + GCS, 2 children—MMF + GCS, 2 children—CsA + AZA + GCS, 1 child—CsA + MMF + GCS.

The control group consisted of 18 healthy children (12 girls, 6 boys; mean age 8.40 ± 3.87 years), diagnosed for primary nocturnal enuresis or suspected urinary tract abnormalities, which were finally excluded.

Blood and urine were collected once in the control group and twice in the children with INS: at disease onset and immediately after remission was achieved. Blood samples were drawn from cubital vein after an overnight fast, during routinely performed laboratory tests. Samples were clotted for 30 min and then centrifuged at room temperature for 15 min. Urine samples were collected on the same day as blood samples. After centrifuging them for 15 min, sediment was removed. Biological material was stored frozen at −70 °C until assayed. Serum and urine Hpx, serum creatinine, albumin, total cholesterol and CRP (C-reactive protein) levels, and urine creatinine and protein concentrations were determined in all children. In all enrolled patients, inflammatory parameters were negative and renal function was normal (creatinine was determined by enzymatic method, the estimated GFR calculated according to the Schwartz formula [36]). Proteinuria was assessed by the urinary protein creatinine ratio (uPCR) on a first morning urine sample. A uPCR > 2 (2 mg/mg) was assumed as the value defining nephrotic proteinuria [2]. Other biochemical tests were determined by standard laboratory methods using an Olympus 5800 analyzer.

Hemopexin concentrations in serum (sHpx) and urine (uHpx) were determined by ELISA using commercial assays according to the manufacturer’s instructions (AssayPro, St. Charles, MO, USA, kit catalog number for serum assays: EH1001-1, for urine assays: EH2001-1). The evaluations were performed twice and then the average of obtained results was calculated. Hpx values were expressed in ng/mL. The sensitivity of the method was 50 ng/mL and 4 ng/mL for sHpx and uHpx, respectively.

Kidney biopsy was performed in 18 children with INS with frequent relapses: MCD was diagnosed in 3 children, FSGS in 7, and MGN in 8. In the remaining patients with a good therapeutic response to GCS, MCD was diagnosed empirically, which is in line with the current recommendations. Histopathological examinations of kidney biopsies were carried out and assessed at the Department of Patho-morphology and Oncological Cytology of the Medical University in Wroclaw. Histopathological analysis included light microscopy, histochemical and immunohistochemical examinations.

The study was conducted according to the guidelines of the Declaration of Helsinki, and approved by the Bioethics Committee of the Wroclaw Medical University (No. KB—199/2009). Informed consent was obtained from the parents and subjects above 16 years old.

## 4. Statistical Analysis

The results are expressed as median values and interquartile ranges. Due to the small number of patients, verification of the hypothesis of median value equality in regard to studied parameters in individual groups was carried out using the non-parametric Kruskal-Wallis rank sum test. Verification of the hypothesis of median value equality in regard to the studied parameters in individual dependent samples (e.g., relapse–remission) was conducted using non-parametric Wilcoxon pair sequence test. Relations between parameters were defined by Pearson’s correlation coefficient r. A *p* value < 0.05 was considered statistically significant. The statistical analysis was performed using a software package EPIINFO Ver. 7.1.1.14 Centers for Disease Control and Prevention (CDC), Atlanta, GA, USA (dated 2 July 2013). The results are presented in the tables and figure.

## 5. Results

All examined parameters in controls were within the normal range.

The data of basic biochemical parameters in children with INS are shown in the table below (Table 1). A significantly higher level of proteinuria during relapse was shown in children requiring additional immunosuppressive treatment compared to the group treated only with GCS.

**Figure 1:**
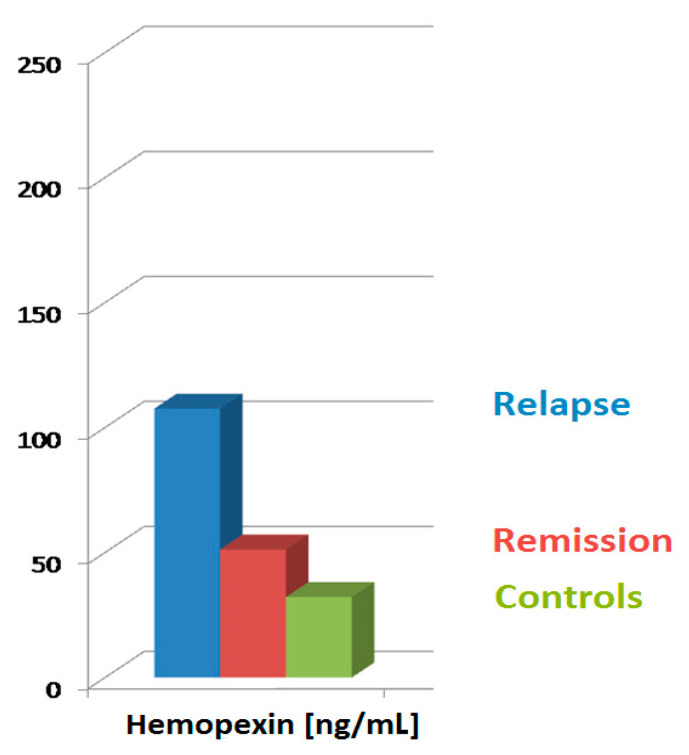
sHpx levels according to clinical phase of the disease, compared to control group. sHpx, serum hemopexin.

sHpx and uHpx levels in the whole INS group were significantly higher, both in relapse and remission, compared to the controls. Additionally, sHpx and uHpx values in relapse were increased compared to those in remission (Table 2, Figure 1).

Analyzing the group of children with the first manifestation of the disease and the group of children with subsequent relapses, we did not show a significant difference in the concentration of sHpx, both in relapse and in remission. uHpx excretion was comparable in the acute phase of the disease in both groups.

On the other hand, children with the first onset of the disease shortly after reaching remission demonstrated lower uHpx values than those with subsequent remission (Table 3).

There was no difference in sHpx levels between the group receiving only GCS (group IIA) and children receiving GCS and additional immunosuppressants (group IIB), both in the relapse and in the remission. However, there was a difference in uHpx values between these groups. During the relapse uHpx was significantly higher, and then in the remission—lower in children receiving only GCS (Table 4).

Correlation analysis performed in the whole group of INS children in relapse did not show any relationship between the sHpx and uHpx levels and CRP, albumin, total cholesterol or proteinuria.

The analysis of sHpx and uHpx concentrations in the acute phase of the disease did not show any significant statistical difference between the groups depending on the histopathological diagnosis. However, in remission, a statistically significant lower median concentration of uHpx was demonstrated in the group of children diagnosed with MCD compared to the group with MGN (*p* = 0.01). However, due to the small number of patients in the analyzed subgroups, the above observations have not been fully presented in our current study.

## 6. Discussion

The involvement of circulating factors in the pathogenesis of nephrotic syndrome in children, not associated with mutation of genes encoding podocyte or basement membrane proteins, has long been discussed [37,38,39]. Experimental results have suggested that one of these factors may be hemopexin, existing in plasma in an inactive form. The active form of Hpx exhibits serine protease properties and can affect podocyte function and structure [40]. Studies in rats have shown, among other facts, that administration of recombinant Hpx to one kidney results in a reversible, massive proteinuria and morphological changes, similar to those observed in MCD, with a loss of negative charge of the glomerular filtration barrier [41]. Lennon et al. demonstrated that Hpx induces nephrin-dependent reorganization of the cytoskeleton of podocytes by rearranging the actin in their cytoskeleton and reducing the glycocalyx [42]. The authors also found that preincubation of podocytes with plasma from healthy humans significantly reduced the degree of cytoskeletal reorganization after Hpx administration, suggesting that, in patients with MCD lesions, protective plasma factors may be lost, rendering podocytes vulnerable to active Hpx. The aim of our study was to show whether the Hpx levels in serum and urine change in children with INS, which would indirectly indicate their role in the induction of nephrotic proteinuria. In this study, the whole INS group showed significantly increased levels of sHpx and uHpx in the relapse, compared to the control group. In remission, the sHpx and uHpx values decreased, but they were still significantly higher than in healthy children, which may suggest the influence of Hpx on the induction of proteinuria. In our study, we also attempted to measure Hpx in children with nephrotic proteinuria in the course of other glomerulopathies. Our preliminary research showed increased sHpx and uHpx concentrations during the relapse and the remission, and these values were not statistically different from those in the group of children diagnosed with INS (results not published). However, it was a very heterogeneous and small group (10 children) and for this reason, we did not include this group in this study. Despite the fact that our observation is based on a small group of children, it seems interesting in the context of further research on the role of Hpx in the development of proteinuria, not only in INS but also in children with other glomerulopathies.

In the literature, data on this subject are scarce. The results of the only study are not entirely consistent with our observations. Bakker et al., who studied nephrotic syndrome due to MCD in children, showed reduced plasma Hpx levels compared to controls [38]. The lowest values were noted in the relapse [43]. These discrepancies are probably due to the use of different methods for Hpx determination and, in addition, the control group was numerically small and differed in age from the study group. The rocket electrophoresis method was used to determine the plasma Hpx titer using anti-Hx IgG. The authors emphasized that plasma titers could be underestimated due to changed Hpx configurations. On the other hand, the control group of 10 subjects consisted of adults up to 35 years of age, whose Hpx titers did not differ from two control samples of children, whose age was not given. It is worth reminding that serum Hpx level changes with age: in neonates this value constitutes about 20% and in children about 80% of the adult value (i.e., 0.4–1.5 g/L) [44,45,46]. The fact that children in remission constituted a different group than those in relapse, as well as a lack of data concerning the time when the samples were taken from the moment of achieving remission, also raises doubts. The methods of statistical analysis were not provided either. Regarding urinary Hpx levels, the authors only report that their values in patients in the relapse were higher than in remission, which is consistent with our results, but lower compared to the controls. However, the lack of data on the statistical significance of the differences does not allow us to interpret the results or to compare them with our results. The authors explain the decrease in plasma Hpx levels in the acute phase of MCD by a possible change in the configuration of the Hpx molecule into an isoform exhibiting protease activity. In fact, they found a marked increase in it by examining the expression of ecto-apyrase, an indicator of glomerular extracellular matrix damage, after prior incubation of kidney sections in plasma from children with nephrotic range proteinuria. In contrast, protease activity was not demonstrated after using plasma from patients with MCD in remission and plasma from healthy subjects. What is the explanation for the increase of Hpx concentration in MCD? Hpx is mainly produced by the liver as an acute phase protein after stimulation with post-inflammatory cytokines, including IL-1 and IL-6, and reflects activation of the inflammatory cascade [47]. In this study no correlation between Hpx and CRP was found, which does not exclude its hepatic synthesis by other inflammatory factors. Indeed, IL-6 levels have been shown to increase in MCD relapse, both in adults and children [48,49].

Hpx can also be partially released into the circulation from glomerular mesangial cells, as evidenced by the observations of Kapajos et al. [50]. Indeed, they demonstrated the presence of Hpx in the supernatant of glomerular mesangial cells collected from healthy individuals after prior incubation with TNF-alpha. It is likely that the local production of Hpx by these cells also acts directly on the glomerular filtration barrier, including podocytes. In our study, the fact that shortly after reaching remission Hpx levels, although lower than in relapse, do not normalize, indicates that pathological processes have not been fully silenced.

This is a premise for the continuation of treatment. From the previously presented experimental studies, it is clear that Hpx affects the permeability of the glomerular filtration barrier and leads to proteinuria. Therefore, one would expect a relationship between the tested glycoprotein and proteinuria. However, no such correlation was demonstrated. This may be due to the small size of the study group. The relationship between serum Hpx and proteinuria was demonstrated by Krikken et al. in their study of 557 renal transplant recipients at risk of graft loss. They demonstrated significantly higher proteinuria in patients with higher plasma Hpx levels and more rapid dysfunction of the transplanted kidney compared with patients with lower Hpx values. Although multivariate analysis showed that this glycoprotein is also an independent risk factor for kidney allograft loss, according to the authors increased permeability of glomerular filtration barrier to proteins is important; proteinuria is a recognized modifiable factor in the progression of chronic kidney disease [51].

Considering the division of patients according to the number of relapses (first occurrence of INS vs. subsequent relapses of INS, treated with GCS), it was shown that serum Hpx levels in relapse and remission are similar, whereas urinary Hpx levels in remission are significantly higher in children with subsequent relapses compared to patients with first episode of INS. To the best of our knowledge, this is the first observation of this kind, probably related to shorter intervals between relapses, which are not sufficient to silence the disorganization of the glomerular filtration barrier. Similar observations were made by Pukajło and Zwolińska, who studied the same groups of children with INS in relation to IL-13—a circulating factor considered a significant enhancer of glomerular permeability in MCD [52].

Taking into account the division according to a more or less intensive therapy, it was demonstrated that serum Hpx concentration, both in relapse and remission, is similar in both groups, contrary to its urinary values. Urinary Hpx excretion was significantly higher in relapse than in remission in children treated only with GCS. This is also the first observation of this kind. Perhaps, during relapse in the group receiving additional immunosuppressive drugs, it is related to a greater suppression of immunocompetent cells with a subsequent decrease in the production of cytokines involved in the pathogenesis of MCD, according to the “two-hit” theory. In turn, increased uHpx in remission could be explained by the persistence of higher local levels of these cytokines in children receiving combination therapy. This hypothesis is supported, among other factors, by the higher urinary IL-13 levels in this group of patients during remission; however, it should be emphasized that they are significantly lower in relation to the values in relapse [52]. 

Correlation studies on Hpx and biochemical markers of nephrotic syndrome did not show significant associations in the whole group of patients.

As mentioned above, the expected correlation between proteinuria and Hpx was not found in any of the study groups. The explanation of this fact may be, apart from the small number of patients, the influence of other circulating factors, including cytokines, which interact leading to one goal. The link between Hpx and the cytokine network is supported by the results of numerous studies, concerning inflammatory processes, including sepsis [53,54], as well as the aforementioned observations presented by Kapajos et al. [45].

This study has several limitations. Firstly, it is a single center study and the group size is limited. We are aware that the number of children in the control group is relatively small. However, it should be noted that small differences in values of Hpx, both in the serum and urine, between the healthy subjects were shown. Secondly, the subgroup receiving combination therapy is rather heterogenic. However, pediatric nephrologists are familiar with the difficulty of selecting a homogeneous group among children with steroid-dependent and frequent-relapsing INS, requiring alternative treatment. Third, our study is a retrospective study. A long-term prospective study would be necessary to confirm our suggestion concerning the role of Hpx as a disease predictor. It would be very valuable to compare the dynamics of Hpx concentrations at the first onset of the disease and at each subsequent relapse in a single patient, and then to analyze the results in groups depending on the number of relapses. We hope that our current results will encourage the programming of further research in this area.

## 7. Conclusions

The increased sHpx and uHpx levels in relapse of INS, as well as their significant decrease in remission, suggest the role of this circulating factor in the pathogenesis of nephrotic range proteinuria. Maintenance of elevated sHpx and uHpx values just after reaching remission speak to the persistence of immune system activation and the need for further treatment. Increased uHpx concentration in children with subsequent relapses, when compared to patients with the first INS episode, may be a useful prognostic marker of the course of INS and an indicator for continuation of immunosuppressive treatment.

Further prospective studies are needed to confirm our results and to concern Hpx as a target for alternative therapy. We are aware that the pathogenesis of INS is complex and that new factors involved in this process are constantly being sought. Our work is part of the research on new pathogenetic links that may enrich therapeutic solutions in the future.

## Figures and Tables

**Table 1:** Selected biochemical parameters in all children with INS and in examined subgroups according to the number of relapses and treatment modality. Data are presented as median values and interquartile ranges.

Parameter Group	Serum Albumin [g/dL]	Total Cholesterol [mg/dL]	Protein/Creatinine Ratio [g Protein/g Creatinine]	CRP [mg/L]
Total INS *N* = 51	1.90 (1.05–2.55)	363.0 (268.0–475.0)	6.2 (3.0–10.6)	2.90 (0.80–3.60)
Group IA *N* = 20	1.70 (1.40–2.20)	372.0 (297.0–464.0)	4.85 (2.40–7.90)	1.75 (0.40–3.60)
Group IB *N* = 31	2.40 (1.00–3.10)	329.5 (238.0–601.0)	7.0 (3.9–10.7)	3.10 (1.65–3.69)
Group IIA *N* = 26	1.90 (1.10–2.50)	366.5 (286.5–442.5)	4.71 (2.5–7.76) ^a^	3.30 (1.40–3.60)
Group IIB *N* = 22	2.0 (1.00–2.50)	329.5 (264.0–636.0)	9.6 (6.2–19.2)	2.56 (1.60–4.20)

^a^—group IIA versus group IIB, *p* = 0.023, Kruskal-Wallis test. INS, idiopathic nephrotic syndrome; CRP, C-reactive protein; Group IA- children with the first occurrence of INS; Group IB- children with relapses of INS; Group IIA- children treated only with GCS, gluco-corticosteroids; Group IIB- children treated with GCS and corticosteroid-sparing agents.

**Table 2:** Serum and urine Hpx levels in the whole group of INS children, depending on disease clinical phase compared to the control group. Data are presented as median values and interquartile ranges.

Group Parameter	Relapse	Remission	Control
sHpx [ng/mL]	107.6 (97.2–114.0) ^a,b^	51.2 (46.4–55.6) ^a^	32.2 (30.8–33.6)
uHpx [ng/mL]	62.8 (55.3–128.0) ^a,b^	27.5 (22.9–31.1) ^a^	15.8 (14.4–17.5)

^a^—relapse INS versus control group and remission INS vs. control group (sHpx, uHpx), *p* = 0.0000, Kruskal-Wallis test. ^b^—relapse INS versus remission INS (sHpx, uHpx), *p* = 0.00000, Wilcoxon test. Hpx, hemopexin.

**Table 3:** sHpx and uHpx levels in INS subgroups according to the number of relapses (group IA—the onset of disease, IB—subsequent relapses). Data are presented as median values and interquartile ranges.

Group Parameter	IA Relapse *N* = 20	IB Relapse *N* = 31	IA Remission *N* = 9	IB Remission *N* = 26
sHpx [ng/mL]	108.8 (104.0–115.8)	105.2 (96.0–114.0)	46.4 (42.0–58.0)	51.2 (49.2–53.6)
uHpx [ng/mL]	61.8 (57.1–148.0)	71.4 (55.2–121.4)	24.2 (21.7–26.0) ^a^	28.2 (24.8–33.2)

^a^—group IA versus group IB, *p* = 0.006.

**Table 4:** sHpx and uHpx levels in the patient groups according to the treatment modality (group IIA—only GCS, IIB—GCS with immunosuppressive sparing agents). Data are presented as median values and interquartile ranges.

Group Parameter	IIA Relapse *N* = 26	IIB Relapse *N* = 22	IIA Remission *N* = 17	IIB Remission *N* = 17
sHpx [ng/mL]	108.2 (97.2–123.6)	103.4 (95.6–108.8)	51.2 (42.0–55.6)	51.2 (49.2–53.6)
uHpx [ng/mL]	103.0 (57.2–158.0) ^a^	61.1 (55.2–81.4)	24.8 (22.1–27.5) ^b^	31.1 (27.5–35.0)

^a^—group IIA vs. IIB, *p* = 0.026; ^b^—group IIA vs. IIB, *p* = 0.0017.

## Data Availability

Not applicable.
